# Supplementary material for: Calcium bursts allow rapid reorganization of EFhD2/Swip-1 cross-linked actin networks in epithelial wound closure
Source: Nat Commun. 2022 May 6;13:2492. doi: 10.1038/s41467-022-30167-0 (PMC9076686; doi:10.1038/s41467-022-30167-0)
Supplement: Supplementary file 1 — Supplementary Information [file 41467_2022_30167_MOESM1_ESM.pdf]

Supplementary Information

Calcium bursts allow rapid reorganization of EFhD2/Swip-1 crosslinked actin networks in epithelial wound closure

Franziska Lehne[1], Thomas Pokrant[2], Sabnam Parbin[3], Gabriela Salinas-Riester[3], Jörg Großhans[4], Katja Rust[1], Jan Faix[2] and Sven Bogdan[1]*

1 Institute of Physiology and Pathophysiology, Dept. of Molecular Cell Physiology, Philipps-University Marburg, Germany

2 Institute for Biophysical Chemistry, Hannover Medical School, Germany

3 NGS- Integrative Genomics Core Unit 1, Department of Human Genetics, University Medical Center Gö ttingen, Germany

4 Fachbereich Biologie, Philipps-University Marburg, Germany

*) to whom correspondence should be addressed
sven.bogdan@staff.uni-marburg.de

a

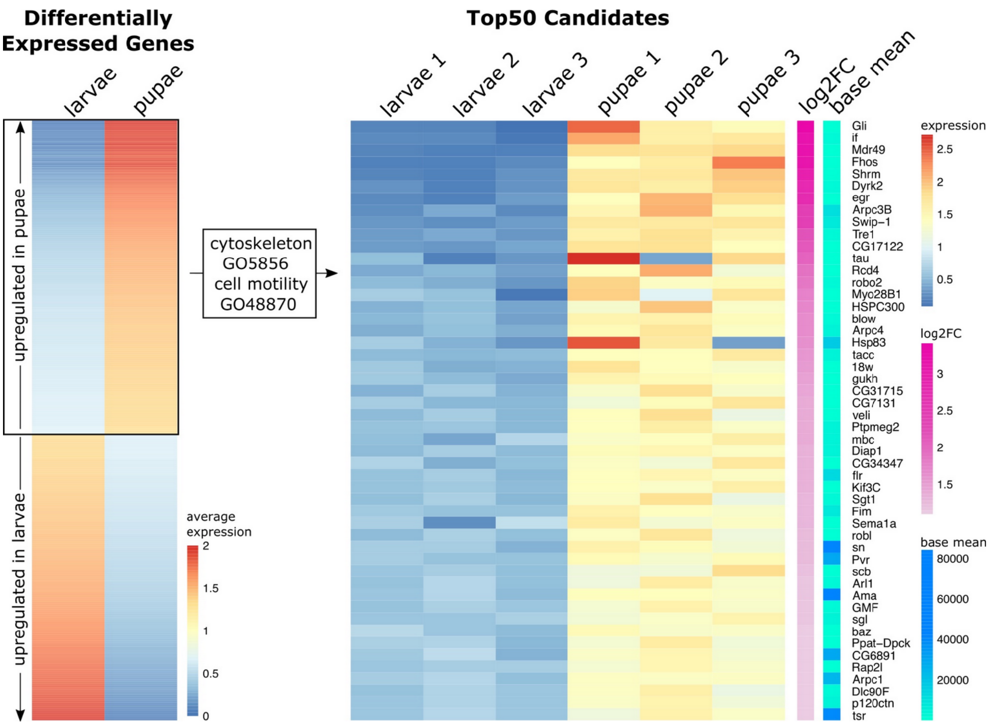

b

flybase gene ID	gene name	known/predicted molecular function	log2FC
FBgn0266084	*fhos*	actin filament bundling/nucleator	3.16
FBgn0085408	*shrm*	actin filament binding	2.97
FBgn0065032	*arpc3B*	Arp2/3 subunit	2.46
FBgn0032731	*swip-1*	actin filament binding	2.46
FBgn0028371	*jbug*	filamin	2.31
FBgn0040299	*myo28B1 (myoVIIB)*	motor protein	1.75
FBgn0061198	*HSPC300*	WRC subunit	1.71
FBgn0284255	*arpc4*	Arp2/3 subunit	1.65
FFBgn0051715	*CG31715*	actin filament capping	1.46
FBgn0260049	*Flr (aip)*	actin filament binding	1.33
FBgn0024238	*fim*	actin filiament crosslinking	1.28
FBgn0003447	*sn*	actin filament crosslinking	1.25

c

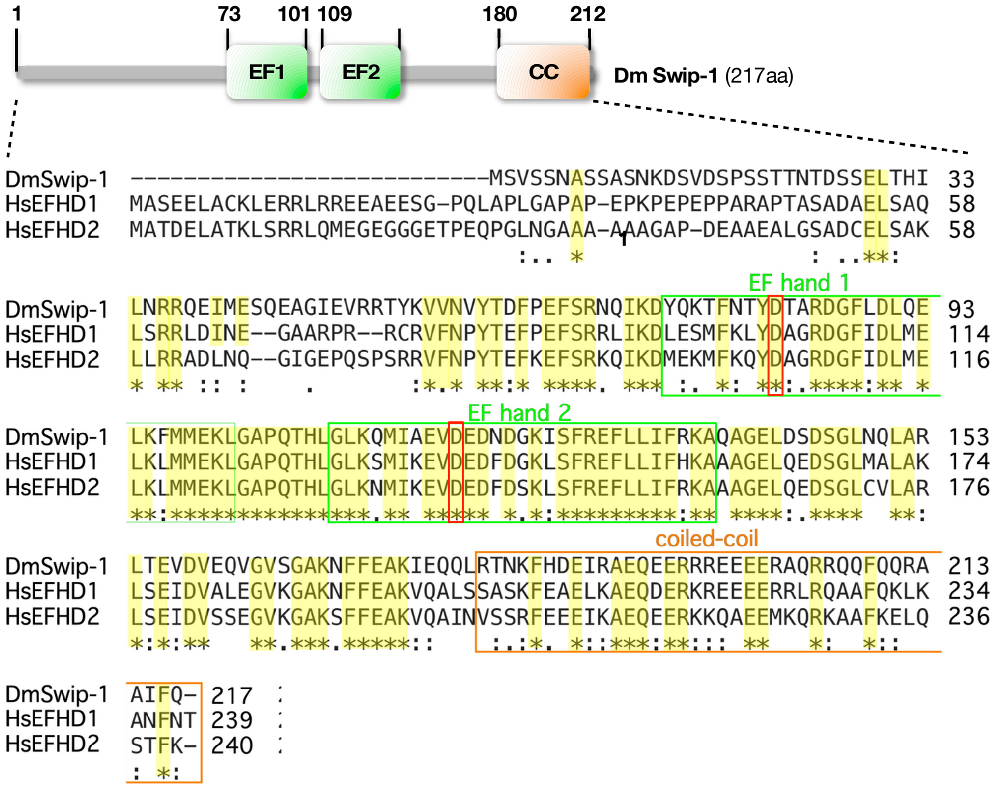

Supplementary figure S1: Swip-1 – a pro-migratory gene conserved between flies and humans

(a) Heatmap showing average expression of all differentially expressed genes in larval and prepupal hemocytes. We selected genes in the GO-terms cytoskeleton (GO:0005856) and cell motility (GO:0048870) in order to identify novel pro-migratory candidates. The expression per replicate, log2FoldChanges and base means of the 50 genes with the highest log2FoldChange, upregulated in prepupaeare shown on the right. **(b)** Selected up-regulated cytoskeletal genes with Fold-changes of larval versus prepupal macrophages. **(c)** Schematic of the *Drosophila* EFhD2/Swip-1. A sequence alignment between Drosophila EFhD2/Swip-1 and the two known human homologues, EFhD1/Swip-2 and EFhD2/Swip-1 is shown. The conserved EF domains and the subsequent coiled-coil regions (orange) are highlighted. The highly conserved aspartate residues of both EF-loops mutated in EFhD1/Swip-1-D82A/D118A are marked by red boxes. Identical residues are marked by an asterisk.

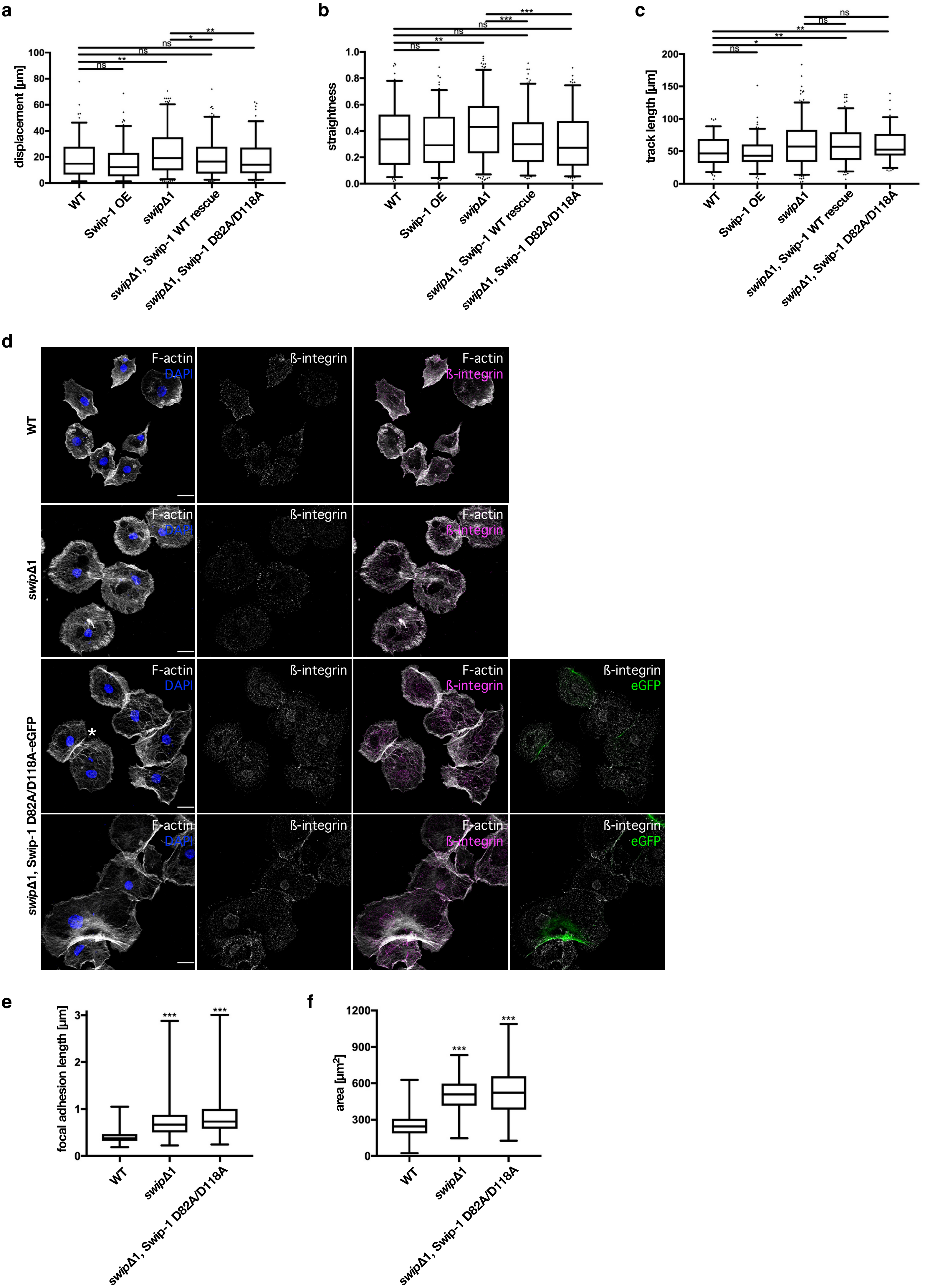

a
displacement [µm]
WT
Swip-1 OE
swipΔ1
swipΔ1, Swip-1 WT rescue
swipΔ1, Swip-1 D82A/D118A
b
straightness
c
track length [µm]
d
F-actin
DAPI
ß-integrin
eGFP
swipΔ1, Swip-1 D82A/D118A-eGFP
e
focal adhesion length [µm]
f
area [µm²]

Supplementary figure S2: Loss of Swip-1 impairs cell migration and focal adhesion turnover of macrophages

(a) Quantification of track displacement, **(b)** track straightness calculated as the ratio of track displacement to track length and **(c)** track length. n= WT: 114, OE: 133, swip-1 mutant: 206, rescue WT: 166, rescue Swip-1-D82A/D118A: 136 tracks. Boxes indicate 50% (25-75%) and whiskers (5-95%) of all measurements, with black lines depicting the medians. For statistical analysis, two values indicated by connecting black lines were compared with two-sided Mann–Whitney test, P value: 0.12 (ns), 0.033 (*), 0.002 (**), <0.001 (***). **(d)** Maximum intensity projection of confocal images of pupal wild-typic, mutant and mutant macrophages re-expressing GFP-tagged calcium binding mutant Swip-1 variant under the control of the hemolectin-Gal4 driver plated on vitronectin coated cover slips. Scale bars represent 10 µm. Cells were co-stained with anti-ß-integrin antibody (white/magenta), DAPI (blue) and phalloidin (white). The asterisk marks a contact site of two macrophages enriched in ß-integrin staining and Swip-1-D82A/D188A-eGFP localization. Images shown are representative of two independent experiments. **(e)** Length of randomly chosen focal adhesions (FA) marked by anti-ß-integrin staining in the lamellipodia were measured in a single z-plane using Image J. n= WT: 443 FA of 26 macrophages, swip-1 mutant: 645 FA of 41 macrophages, calcium-binding mutant Swip-1-D82A/D118A: 573 FA of 35 macrophages. **(f)** Cell size of single wild type, mutant and mutant macrophages re-expressing GFP-tagged calcium binding mutant Swip-1 variant was measured using Image J Analyze particles macro. n= WT: 65, swip-1 mutant: 60, calcium-binding mutant Swip-1-D82A/D118A: 59 macrophages. (e, f) Boxes indicate 50% (25-75%) and whiskers minimum and maximum of all measurements, with black lines depicting the medians. Two-sided Mann–Whitney test was used, P value: <0.001 (***).

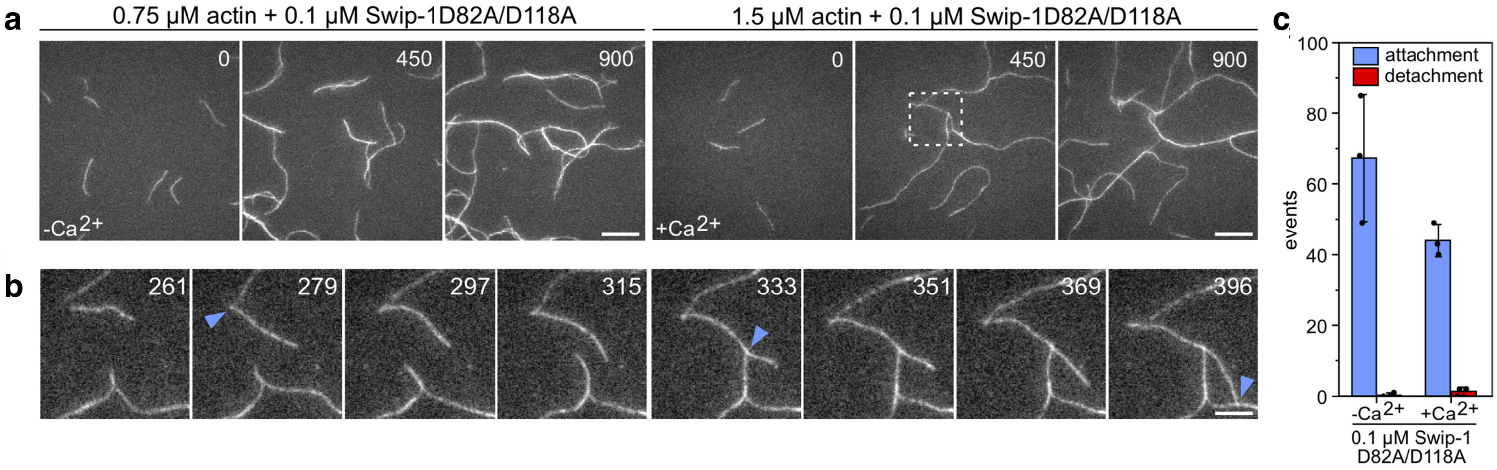

Supplementary figure S3: *Drosophila* Swip-1-D82A/D118A maintains stable cross-links in the presence of calcium.

(a) Time-lapse micrographs of TIRFM assays, used for analysis of cross-linking behavior of the Swip-1-D82A/D118A EF-hand mutant in the absence or presence of 1 mM Ca^{2+}. Polymerization of 0.75 or 1.5 µM G-actin (10% ATTO488-labelled) with 0.1 µM Drosophila Swip-1 D82A/D118A in the absence and presence of Ca^{2+} as indicated. Note significantly cross-linked networks in the absence of Ca^{2+} as compared to assay conditions in the presence of Ca^{2+}. **(b)** Enlarged gallery of inset (dashed box in a) displays stable cross-linking behavior of 0.1 µM Drosophila Swip-1 D82A/D118A in the presence of Ca^{2+} at higher temporal resolution. Blue arrowhead marks a newly formed cross-link. Time is given in seconds in the upper right corner of each frame. Scale bars, 10 µm. **(c)** Quantification of attachment and detachment event in the absence or presence of Ca^{2+}. Bars represent mean ± SD from three movies each (n=3). Data points show the measured values of the experiments.
