# Supplementary material for: Calcium bursts allow rapid reorganization of EFhD2/Swip-1 cross-linked actin networks in epithelial wound closure
Source: Nat Commun. 2022 May 6;13:2492. doi: 10.1038/s41467-022-30167-0 (PMC9076686; doi:10.1038/s41467-022-30167-0)
Supplement: Supplementary file 3 — Description of Additional Supplementary Files [file 41467_2022_30167_MOESM3_ESM.pdf]

Description of Additional Supplementary Files

File Name: Supplementary Movie 1
Description: **Swip-1 localizes to protruding lamellipodia**
Random migration of macrophages expressing EFhD2/Swip-1-eGFP transgene imaged in living wild type pupa (4h APF). Note: Some cells migrate along tracheae. Scale bar 10 µm.

File Name: Supplementary Movie 2
Description: **Loss of Swip1 speeds up 3D cell migration of macrophages**
Spinning-disk microscopy videos of randomly migrating macrophages in prepupae (4h APF) with indicated genotypes were tracked by using Imaris 9.3. Track speed mean is color-coded (0.06 to 6µm/min).

File Name: Supplementary Movie 3
Description: **Calcium binding of Swip-1 is required for localization and function**
Random migration of macrophages expressing Swip-1-D82A/D118A-eGFP transgene imaged in living wild type pupa (4h APF). Note: Some cells migrate along tracheae. Scale bar 10 µm.

File Name: Supplementary Movie 4
Description: **EFhD2/Swip-1 is a cross-linking protein.**
Representative time-lapse TIRFM movies of the assembly of actin filaments in the presence of Drosophila Swip-1, human EFhD2, -actinin4 or fascin. 1.0 µM G-actin (10% ATTO488 labeled) was polymerized either in the presence of 1.0 µM Drosophila Swip-1, 500 nM human EFhD2, 500 nM -actinin4 or 500 nM fascin. As opposed to EFhD2, -actinin and fascin, which formed prominent bundles, Drosophila Swip-1 did not induce formation of bundles, but exclusively promoted the formation of cross-linked networks. Notably, however, EFhD2 also exhibited cross-linking activity. Time is in min:sec. Scale bar 15 µm.

File Name: Supplementary Movie 5
Description: **Swip-1 forms stable cross-links in the absence of Ca^{2+}.**
Representative time-lapse TIRFM movies of the assembly of actin filaments in the absence of Ca^{2+}. 0.75 µM G-actin (10% ATTO488 labeled) was polymerized either in the absence (control, left panel) or presence of 0.1 µM (middle panel) or 1.0 µM Drosophila EFhD2/Swip-1 (right panel). Even at 0.1 µM, Swip-1 formed stable cross-links between elongating actin filaments, creating a stable and highly cross-linked network, while actin control filaments did not form any cross-linked arrays. Note the almost perpendicular arrangement of cross-links formed by Drosophila EFhD2/Swip-1. Time is in min:sec. Scale bar 15 µm.

File Name: Supplementary Movie 6
Description: **Bundles formed by EFhD2 remain stable in the presence of Ca^{2+}.**
Representative time-lapse TIRFM movies of the assembly of actin filaments in the absence or presence of Ca^{2+}. 0.75 µM or 1.5 µM G-actin (10% ATTO488 labeled) was polymerized in the presence of 1.0 µM human EFhD2. Notably, bundling activity was not affected by calcium. Time is in min:sec. Scale bar 15 µm.

File Name: Supplementary Movie 7
Description: **Swip-1 forms transient cross-links in the presence of Ca^{2+}.**

Representative time-lapse TIRFM movies of the assembly of actin filaments in the presence of Ca^{2+}. 1.5 µM G-actin (10% ATTO488 labeled) was polymerized either in the absence (control, left panel) or presence of 0.1 µM (middle panel) or 1.0 µM Drosophila EFhD2/Swip-1 (right panel). Cross-links between actin filaments (not seen with the control) were rare in the presence of Ca^{2+} at the low Drosophila EFhD2/Swip-1 concentration of 0.1 µM. At 1.0 µM Drosophila EFhD2/Swip-1 the crosslinks were highly dynamic and short lived as seen by frequent detachment of crosslinked filaments. Time is in min:sec. Scale bar 15 µm.

File Name: Supplementary Movie 8
Description: **Human EFhD2 forms transient cross-links in the presence of Ca^{2+}.**
Representative time-lapse TIRFM movies of the assembly of actin filaments in the absence or presence of Ca^{2+}. 0.75 µM or 1.5 µM G-actin (10% ATTO488 labeled) was polymerized in the presence of 0.1 µM human EFhD2. In variation to Drosophila Swip-1, EFhD2 exhibited both bundling and cross-linking activity. Note that cross-linking virtually disappeared in the presence of calcium. Time is in min:sec. Scale bar 15 µm.

File Name: Supplementary Movie 9
Description: **Calciumbinding mutant Swip-1-D82A/D118A forms stable cross-links and bundles even in the presence of Ca^{2+}.**
Representative time-lapse TIRFM movies of the assembly of actin filaments in the absence or presence of Ca^{2+}. 0.75 or 1.5 µM G-actin (10% ATTO488 labeled) were polymerized in the presence of 0.1 µM Swip-1-D82A/D118A. Cross-links between actin filaments were stable even in the presence of Ca^{2+}. Time is in min:sec. Scale bar 15 µm.

File Name: Supplementary Movie 10
Description: M10: **An in vivo single-cell wounding model of the abdominal epidermis in the early Drosophila pupa**
Spinning-disk microscopy video of the abdominal epidermis from a wild type 18h APF old pupa ubiquitously expressing a Lifeact-eGFP transgene under the control of the da-Gal4 driver. Images were taken every 30 sec for 60 min, ablation starts at t=0 min. Scale bar 25 µm.

File Name: Supplementary Movie 11
Description: **Swip-1 is immediately recruited to lamellipodial protrusions upon wounding**
Spinning-disk microscopy video of the abdominal epidermis from a wild type 18h APF old pupa ubiquitously expressing a EFhD2/Swip-1-eGFP transgene under the control of the da-Gal4 driver. Images were taken every 20 sec for 30 min, ablation starts at t=0 min. Scale bar 25 µm.

File Name: Supplementary Movie 12
Description: **Single-cell wounding response depends on calcium waves**
Spinning-disk microscopy video of the abdominal epidermis from a wild type 18h APF old pupa ubiquitously expressing the calcium indicator RCaMP under the control of the da-Gal4 driver. Images were taken every 250 msec for 72 sec in total, ablation starts at t=0 sec. Changes of intracellular calcium are visible as an increasing fluorescence intensity. The calcium wave is propagated in a circular fashion with a velocity of 2.2 ± 0.7 µm/sec. Scale bar 25 µm.

File Name: Supplementary Movie 13
Description: **Epithelial wound closure requires Swip-1 function**

Spinning-disk microscopy video of the abdominal epidermis from a swip-1 mutant 18h APF old pupa ubiquitously expressing a Lifeact-eGFP transgene under the control of the da-Gal4 driver. Images were taken every 30 sec for 60 min, ablation starts at t=0 min. Scale bar 25 µm.

File Name: Supplementary Movie 14
Description: **Loss of mouse EFhD2 in B16-F1 cells impairs 2D cell migration.**
Random migration of B16-F1 and derived EFhD2-KO cells (clone #10) on laminin. Cells were recorded by phase-contrast, time-lapse imaging using a 4x with objective with additional optovar 1.6x magnification and tracked by MTrackJ to illustrate representative cell trajectories. Note the shorter travelled distance by the mutant cells. Time is indicated in h:min:sec. Scale bar 100 µm.

File Name: Supplementary Movie 15
Description: **Activated Arp2/3 complex and Swip-1 induce formation of cross-linked dendritic actin networks.**
Representative time-lapse TIRFM movie of the assembly of actin filaments in the presence of active Arp2/3 complex and Swip-1. 0.5 µM actin (10% ATTO488 labeled) was co-polymerized with 0.1 µM Swip-1, 0.25 nM Arp2/3 complex and 5 nM WAVE2-VCA in the absence of Ca^{2+}. Under these conditions, activated Arp2/3 complex formed branches at an angle of ~70° degrees to the respective mother filaments (red arrowheads) while Swip-1 preferentially formed cross-links (cyan arrowheads) at perpendicular angles between spontaneously growing and Arp2/3 complex nucleated filaments to yield a lamellipodium-like network over time. Time is in min:sec. Scale bar 15 µm.
