# Supplementary material for: Calcium bursts allow rapid reorganization of EFhD2/Swip-1 cross-linked actin networks in epithelial wound closure
Source: Nat Commun. 2022 May 6;13:2492. doi: 10.1038/s41467-022-30167-0 (PMC9076686; doi:10.1038/s41467-022-30167-0)
Supplement: Supplementary file 19 — Reporting Summary [file 41467_2022_30167_MOESM19_ESM.pdf]

nature research

Corresponding author(s): Sven Bogdan

Last updated by author(s): 2022/03/31

Reporting Summary

Nature Research wishes to improve the reproducibility of the work that we publish. This form provides structure for consistency and transparency in reporting. For further information on Nature Research policies, see Authors & Referees and the Editorial Policy Checklist .

Statistics

For all statistical analyses, confirm that the following items are present in the figure legend, table legend, main text, or Methods section.

n/a	Confirmed	
☐	☒	The exact sample size (*n*) for each experimental group/condition, given as a discrete number and unit of measurement
☒	☐	A statement on whether measurements were taken from distinct samples or whether the same sample was measured repeatedly
☐	☒	The statistical test(s) used AND whether they are one- or two-sided<br>*Only common tests should be described solely by name; describe more complex techniques in the Methods section.*
☒	☐	A description of all covariates tested
☐	☒	A description of any assumptions or corrections, such as tests of normality and adjustment for multiple comparisons
☐	☒	A full description of the statistical parameters including central tendency (e.g. means) or other basic estimates (e.g. regression coefficient) AND variation (e.g. standard deviation) or associated estimates of uncertainty (e.g. confidence intervals)
☐	☒	For null hypothesis testing, the test statistic (e.g. *F*, *t*, *r*) with confidence intervals, effect sizes, degrees of freedom and *P* value noted<br>*Give P values as exact values whenever suitable.*
☒	☐	For Bayesian analysis, information on the choice of priors and Markov chain Monte Carlo settings
☒	☐	For hierarchical and complex designs, identification of the appropriate level for tests and full reporting of outcomes
☐	☒	Estimates of effect sizes (e.g. Cohen's *d*, Pearson's *r*), indicating how they were calculated

Our web collection on statistics for biologists contains articles on many of the points above.

Software and code

Policy information about availability of computer code

Data collection	Zen Blue 2.5 (Zeiss); Imaris 9.3 (Bitplane); LasX (Leica)
Data analysis	ImageJ 1.51 (NIH); Prism 7 (GraphPad); Microsoft Excel , Origin Pro 2021 (OriginLab)

For manuscripts utilizing custom algorithms or software that are central to the research but not yet described in published literature, software must be made available to editors/reviewers. We strongly encourage code deposition in a community repository (e.g. GitHub). See the Nature Research guidelines for submitting code & software for further information.

Data

Policy information about availability of data

All manuscripts must include a data availability statement. This statement should provide the following information, where applicable:

- Accession codes, unique identifiers, or web links for publicly available datasets
- A list of figures that have associated raw data
- A description of any restrictions on data availability

All data are available within the article, supplementary Information, or from the source data file.

Field-specific reporting

Please select the one below that is the best fit for your research. If you are not sure, read the appropriate sections before making your selection.

☒ Life sciences ☐ Behavioural & social sciences ☐ Ecological, evolutionary & environmental sciences

For a reference copy of the document with all sections, see nature.com/documents/nr-reporting-summary-flat.pdf

Life sciences study design

All studies must disclose on these points even when the disclosure is negative.

Sample size	Sample sizes were chosen on the basis of preliminary phenotypic experiments so as to provide sufficient power for statistical comparison.
Data exclusions	No data were excluded.
Replication	All replicates are reported in the manuscript
Randomization	Randomization was not relevant to this study. Experiments were conducted on different flies or cells on different (random) days
Blinding	Blinding was not relevant for this type of experiments. Researchers allways used controls with defined genotypes.

Reporting for specific materials, systems and methods

We require information from authors about some types of materials, experimental systems and methods used in many studies. Here, indicate whether each material, system or method listed is relevant to your study. If you are not sure if a list item applies to your research, read the appropriate section before selecting a response.

Materials & experimental systems

n/a	Involved in the study	
☐	☒	Antibodies
☐	☒	Eukaryotic cell lines
☒	☐	Palaeontology
☐	☒	Animals and other organisms
☒	☐	Human research participants
☒	☐	Clinical data

Methods

n/a	Involved in the study	
☒	☐	ChIP-seq
☒	☐	Flow cytometry
☒	☐	MRI-based neuroimaging

Antibodies

Antibodies used	anti-ß-integrin (CF.6G11 from DSHB); anti-EFhD2 antibody was kindly provided by Dirk Mielenz, Erlangen
Validation	anti-ß-integrin; link to the website with all relevant information :https://dshb.biology.uiowa.edu/CF-6G11<br>The specificity of antibodies were validated by comparing protein level in wild type versus mutant and/or by the manufactures.<br><br>Informations about Alexa Fluor Antibodies: https://www.abcam.com/

Eukaryotic cell lines

Policy information about cell lines

Cell line source(s)	Drosophila S2R+ cells from DGRC, link to the website with all relevant information: https://dgrc.bio.indiana.edu/product/View?product=150<br>Mouse melanoma cells B16-F1 (ATTC, CRL-6323)
Authentication	Cells were obtained and authenicated by the manufacturers.
Mycoplasma contamination	Cells were confirmed negative for mycoplasma contamination.
Commonly misidentified lines<br>(See ICLAC register)	None of the cell lines used are listed in the ICLAC register.

Animals and other organisms

Policy information about studies involving animals; ARRIVE guidelines recommended for reporting animal research

Laboratory animals	Wound closure experiments were performed using 18-20 h old pupa; macrophages were isolated from white prepupa (0-4h APF). Both, females and males were used for wounding experiments and macrophage isolation. The following fly stocks were obtained from the Bloomington stock center: w[1118] (BL3605), hmlΔ-Gal4 (BL30139), y[*] w[*]; P{w[+mC]=UAS-2xEGFP}AH3 (BL6658), w[*]; P{w[+mC]=UAS-Lifeact.GFP.W}3 (BL57326), y[1] w[*]; P{y[+t*] w[+mC]=UAS-Lifeact-Ruby}VIE-19A (BL35545), w[*]; PBac{w[+mC]=20XUAS-IVS-NES-jRCaMP1a-p10}VK00005 (BL63792). RNAi against EFhD2/Swip-1; w[1118]; P{GD7047}v31307 was obtained from the Vienna Drosophila RNAi Center.

Wild animals	The study did not involve wild animals
Field-collected samples	The study did not involve samples collected from the field
Ethics oversight	Using Drosophila as a model system no ethical approval or guidance was required.

Note that full information on the approval of the study protocol must also be provided in the manuscript.
